# On Hydrated Peroxyde of Iron as an Antidote to Arsenic

**Published:** 1840-01

**Authors:** 


*On Hydrated Peroxyde of Iron as an Antidote to Arsenic.* By a Commission of the Academy of Medicine of Paris.

DOGS were first destroyed by arsenic, to determine the time in which that poison proves fatal when its action is not interfered with, and to obtain a standard by which the beneficial effects of the antidote might be more nearly estimated. Other dogs were killed by ligature of the œsophagus to determine the influence which that necessary operation would have in their destruction. Four different oxides of iron were then used with different dogs to whom arsenic had been given. A moist protoxyde, containing nineteen per cent. of anhydrous peroxyde, and a black oxide containing seven per cent. were entirely inefficacious; the dogs died as soon as they would if no means had been used to save them. The moist hydrated peroxyde, and the common dry hydrated peroxyde of iron (the subcarbonate of iron of former pharmacopœiæ, and ferri-sesqui-oxydum of the present,) both produced more important results. Ten animals poisoned with arsenic were treated with the first. In the first experiments large doses (half a drachm) of arsenic were given, and comparatively small quantities (four, six or eight ounces,) of the magma of peroxyde of iron; yet all the animals lived many hours longer than when the action of the arsenic was left uncontrolled. In the subsequent experiments, twelve grains of arsenic were given, and from eight to sixteen ounces of the antidote; and all the dogs thus treated lived as long as the ligature of the œsophagus would permit them.

With the second compound (the subcarbonate of sesqui-oxyde of iron,) in three experiments, in which four grains of arsenious acid, and three ounces of the antidote were given, the animals, like those last mentioned, lived to the full period which is possible, after tying the œsophagus. The arsenite of peroxyde of iron (the salt which was supposed to be found in the stomach in the preceding cases) was next given to dogs, and evidently acted as a virulent poison. But the apparent anomaly of these cases was proved, by some further experiments, to result from the arsenite of iron, thus administered, being decomposed by the hydrochloric and lactic acids of the gastric secretion, and some uncombined arsenious acid being set free; and this showed that, to counteract the effects of the arsenic it would be necessary to administer the peroxyde of iron in considerable excess, so that every portion of arsenious acid, whether originally existing in the stomach or set free by the decomposition of arsenite of iron, might be completely neutralized.

The results of all these experiments on animals were completely confirmed by the chemical examinations by which M. Guibourt determined the composition and the mutual actions of the substances employed. Thus the first two oxydes that were employed were of no avail against the effects of the arsenic: and from M. Guibourt's chemical researches it was evident that they did not render arsenious acid insoluble. In the physiological experiments the moist hydrated peroxyde of iron neutralised the arsenious acid in the stomach, imperfectly, indeed, when there was a proportionably large quantity of acid, but completely when the proportion of arsenic was small and that of the peroxyde considerable; and in the chemical experiments it appeared that ten parts of the dry peroxyde were necessary to neutralize one of arsenious acid. In the physiological experiments, again, the dry peroxyde completely neutralized the arsenious acid; and in the chemical the same peroxyde reduced to traces scarcely perceptible the precipitate of arsenious acid that could be obtained by Marsh's apparatus.

It is evident then (the Report continues,) that in the circumstances which have been determined, dogs have taken arsenic sufficient to destroy them in a few hours, and that yet they have lived as long as if only the œsophagus had been tied. The poison, therefore, has had no effect;—the peroxyde of iron, which we have employed is, consequently, a real and efficacious antidote to arsenious acid.

Now, it is demonstrated that we have *disempoisoned* dogs that had taken arsenic; we have determined what doses of hydrated peroxyde of iron are necessary to neutralize fatal quantities of arsenious acid; we have employed with the greatest success, not only the moist peroxyde of iron (always an inconvenient compound), but also the dry hydrated peroxyde, so common in the shops under the name of subcarbonate of iron, and so easy to administer in all imaginable doses, and so little injurious that we can see no necessity to limit the employment that may be made of it in these cases of poisoning. The following then, is the mode of treatment which we should advise to be employed in these cases.

The first duty of the physician is to remove from the stomach the greatest possible quantity of the poison by vomiting. For this purpose watery drinks are improper; but while waiting for the peroxyde of iron, the doctor should tickle the vula, and administer oil, which will not dissolve the arsenious acid; but above all, as soon as it can be procured, the patient should be gorged with warm water, charged with some ounces of the peroxyde. The water will cause vomiting, and the peroxyde of iron, which is suspended in it, will neutralize the particles of arsenious acid that are dissolved. This means fulfils at once the two chief indications in every case of poisoning. The antidote must be given as soon as possible, and in such a quantity, and for such a time that there can be no reason to suppose a single atom of arsenious acid remains in the stomach. Four ounces of the dry hydrated peroxyde of iron (the subcarbonate of iron of the shops, the ferri-sesquioxydum of the London Pharmacopœiæ,) should be suspended in twenty-four ounces of water, and a good glass of the mixture should be taken every ten minutes. After four ounces are consumed, fresh doses of the same compound should be administered in the same way, and the patient should not be considered out of danger till he has taken at least half an ounce of the peroxyde for each grain of arsenious acid supposed to have remained in the stomach. If, afterwards, symptoms of inflammation should manifest themselves, prompt recourse must be had to antiphlogistics and other appropriate means.

In the short discussion which followed the reading of the Report, M. Devergie said he thought the conclusion too explicit; that the large quantites of the peroxyde which it was necessary to administer, even when but a small quantity of arsenic had been taken, would always be an obstacle to its use, and that it would be unwise to let the presumed antidote take the place of any of the other means usually resorted to, especially continued vomiting. At the conclusion of the discussion it was agreed to employ the words " antidote" instead of " real and efficacious antidote;" and the Report was then unanimously ordered to be printed.—*Revue Medicale. Mai et Juin*, 1839.—*British and Foreign Med. Rev., July* 1839.

